# Sodium houttuyfonate enhances the mono-therapy of fluconazole on oropharyngeal candidiasis (OPC) through HIF-1α/IL-17 axis by inhibiting cAMP mediated filamentation in *Candida albicans-Candida glabrata* dual biofilms

**DOI:** 10.1080/21505594.2022.2035066

**Published:** 2022-02-23

**Authors:** Mengli Chen, Ting Cheng, Chen Xu, Min Pan, Jiadi Wu, Tianming Wang, Daqiang Wu, Guiming Yan, Changzhong Wang, Jing Shao

**Affiliations:** a Laboratory of Infection and Immunity, College of Integrated Chinese and Western Medicine (College of Life Science), Anhui University of Chinese Medicine, Hefei, Anhui P. R, China; b Department of Anatomy, School of Basic Medicine, Huazhong University of Science and Technology, Wuhan, P. R, China; c Inflammation and Immune Mediated Diseases Laboratory of Anhui Province, School of Pharmacy, Anhui Medical University, Hefei, P. R, China; d Institute of Integrated Traditional Chinese and Western Medicine, Anhui Academy of Chinese Medicine, Hefei, Anhui P. R, China; e Cas Center for Excellence in Molecular Cell Sciences, Ministry of Education Key Laboratory for Membrane-less Organelles & Cellular Dynamics, Hefei National Laboratory for Physical Sciences at the Microscale, School of Life Sciences, Division of Life Sciences and Medicine, University of Science and Technology of China, Hefei, P.r, China

**Keywords:** Candida albicans, *Candida glabrata*, mixed biofilms, hypoxia, sodium houttuyfonate, Oropharyngeal candidiasis

## Abstract

*Candida albicans* and *Candida glabrata* are two common opportunistic fungi that can be co-isolated in oropharyngeal candidiasis (OPC). Hypha is a hallmark of the biofilm formation of *C. albicans*, indispensable for the attachment of *C. glabrata,* which is seldom in mycelial morphology. Increasing evidence reveals a hypoxic microenvironment in interior fungal biofilms, reminding of a fact that inflammation is usually accompanied by oxygen deprivation. As a result, it is assumed that the disaggregation of hypha-mediated hypoxia of biofilms might be a solution to alleviate OPC. Based on this hypothesis, sodium houttuyfonate (SH), a well-identified traditional herbal compound with antifungal activity, is used in combination with fluconazole (FLU), a well-informed synthesized antimycotics, to investigate their impact on filamentation in *C. albicans* and *C. glabrata* dual biofilms and the underlying mechanism of their combined treatment on OPC. The results show that compared with the single therapy, SH plus FLU can inhibit the hyphal growth in the mixed biofilms in vitro, decrease the fungal burden of oral tissues and internal organs, restore mucosal epithelial integrity and function, and reduce hypoxic microenvironment and inflammation in a mice OPC model. The possible mechanism of the combined therapy of SH plus FLU can be attributed to the regulation of HIF-1α/IL-17A axis through direct abrogation of the dual *Candida* biofilm formation. This study highlights the role of HIF-1α/IL-17A axis and the promising application of SH as a sensitizer of conventional antifungals in the treatment of OPC.

## Introduction

*Candida* spp. is an important causative agent that can cause oral fungal infections in especially immunocompromised populationsfor example, HIV and cancer patients [1,2]. Oropharyngeal candidiasis (OPC) is an oral mucosal disturbance featured by a lack of papillae on the back of the tongue, erythema and fissures on the tongue, erythema on the mucosal surfaces, or angular cheilitis [3]. The main pushing hand of OPC is the dimorphic opportunistic fungi *Candida albicans* (>70%) followed by the oval yeast *Candida glabrata* (~10%) with seldom germ tube [4,5]. It has been documented that both *Candida* species become frequently co-isolated in more than 68.2% of the patients with multispecies OPC far beyond the occurrence of other *Candida* spp. [6].

Both *C. albicans* and *C. glabrata* can be co-cultured to form dual biofilms in a competitive manner, different from antagonism between *C. albicans* and *C. krusei* [7]. In their commensalism, the sizes of both *Candida* species are markedly reduced compared with those of mono-cultivation, and the population of *C. albicans* is much lower than that of *C. glabrata* [8]. Although there are controversial observations on the virulence and invasiveness between the dual and the single cultivations of *C. albicans* and *C. glabrata*, the presence of *C. albicans* is deemed to be a facilitator of mucosal colonization and entrance of *C. glabrata* [9]. Actually, it is hard to isolate *C. glabrata* alone from infection niche [10]. Our previous results showed that *C. albicans* and *C. glabrata* dual biofilms were highly resistant to conventional antifungal agents including caspofungin, fluconazole (FLU), and amphotericin B at no expense of subdued virulence compared with each single culture [11,12].

Silva and the colleagues found that *C. glabrata* alone was unable to cause pathological damage in a mouse OPC model, while co-inoculation of *C. albicans* and *C. glabrata* could develop more severe disease than single inoculation of *C. albicans* [13]. Their study further demonstrated that the co-growth of *C. albicans* and *C. glabrata* and the enhanced mucosal penetration of *C. glabrata* rely primarily on the intimate attachment of the yeast form of *C. glabrata* to the thread-like hypha of *C. albicans* [13]. Growing evidence reveals a central role of cAMP in the regulation of filamentation in *C. albicans* [14–16]. Elevated intracellular cAMP concentration promotes the growth of hypha. The filamentation is a prerequisite for the formation of mature *C. albicans* biofilms. As a result, the inhibition of hyphal growth of *C. albicans* might be an effective approach to disorganize *C. albicans-C. glabrata* dual biofilms.

*C. albicans* biofilms are an aggregate of surface-dwelled fungal cells and contain a sophisticated two-layer structure composed of a thin, basal yeast layer and a thick, more open layer comprising profuse matrix and hyphal elements above the basal layer [17,18]. While *C. glabrata* biofilms are a structure of a multilayer with packaged or constituted blastoconidia with scarce pseudohyphae and hyphae [5,19]. The metabolism of interior fungal biofilms is extremely low, partly due to the hypoxic microenvironments, which are considered a typical feature of yeast biofilms and a pivotal factor of antifungal resistance [20,21]. Varying oxygen tension significantly impacts the macroscopic architecture and morphogenesis of fungal biofilms as well as disease progression [22,23]. It is known that infected and inflamed tissues are deprived of oxygen, and the hypoxia-inducible factor 1α (HIF-1α) as a primary sensor of hypoxia helps tissue and immune cells adapt [24]. Increasing clues indicate that the activation of innate Th17 cells and the associated IL-17/IL17R axis are of critical importance in controlling OPC [25–29]. HIF-1α is an indispensable transcription factor in Th17 cell fate determination [30], and regulated by hypoxia, IL‑6 and signal transducer and activator of transcription 3 (STAT3) dependent differentiation signals, driving IL-17 expression followed by forming a complex with retinoic acid receptor-related orphan receptor-γt (RORγt) and histone acetyltransferase p300 [31]. However, the role of HIF-1α in the regulation of IL-17 in response to OPC is still ill-defined. Therefore, we suppose that the biofilm-dependent HIF-1α/IL-17 axis might play a central role in OPC development.

Plant-derived compounds have been used in oral candidiasis for decades [32]. *Houttuynia cordata* Thunb, an edible and perennial herbaceous plant with discernible fishy smell, is mainly distributed in moist and shady areas of East Asian countries, such as China, Japan, and Korea [33]. Houttuynin (decanoyl acetaldehyde, C_12_H_22_O_2_), the major active component of *H. cordata*, is unstable due to its rapid oxidization or polymerization. Sodium houttuyfonate (SH, C_12_H_23_NaO_5_S), an additive product of houttuynin and sodium bisulfite, not only becomes more stable than houttuynin, but also retains the major pharmacological activities of houttuynin, including antifungal, antibacterial, and anti-inflammatory activities [34]. There have been several reports showing a therapeutic effect of *Houttuynia cordata* extract/decoction for oral infectious diseases [35,36]. During the past years, our group has demonstrated the antifungal activity of SH alone and in combination with FLU against planktonic and biofilms of *C. albicans* [37,38]. SH is effective not only in the treatment of systemic candidiasis but also in the therapy of *C. albicans* associated ulcerative colitis (UC) [39,40]. These findings suggest that SH plays a protective role in *Candida* associated mucosal infections.

In this study, the in vitro antifungal activity of SH and/or FLU and their effects on cAMP mediated filamentation are evaluated in *C. albicans* and *C. glabrata* dual biofilms. The therapy of SH and/or FLU and the underlying mechanism associated with HIF-1α/IL-17 axis are also investigated in an OPC mice model caused by the dual *Candida* biofilms.

## Materials and methods

### Strains and growth conditions

*C. albicans* SC5314 and the clinical isolate Z215 were kindly provided by Prof. Yuanying Jiang from College of Pharmacy, the Second Military Medical University (Shanghai, China), and Prof. Huaiwei Lu, Clinical Laboratory, Anhui Provincial Hospital (Anhui, China). *C. glabrata* ATCC15126 and ATCC 28226 were acquired from National Institutes for Food and Drug Control (Beijing, China). The strains were stored at fresh Sabouraud dextrose agar (SDA, 1% peptone, 4% dextrose, and 1.8% agar, m/v) and then subcultured in YPD liquid medium (1% yeast extract, 2% peptone, and 2% dextrose) at 37°C for 12–16 h till the exponential phase. The fungal pellets were harvested at a centrifugation of 3000 g, washed twice with sterile phosphate-buffered saline (PBS, pH 7.2, 0.01 M), and resuspended in RPMI-1640 medium at pH 7.0 modified by 0.165 M MOPS.

### Chemicals

SH was purchased from Kailai Bioengineering (A03148). FLU, cortisone acetate, XTT [(2,3-Bis-(2-methoxy-4-nitro-5-sulfophenyl)-2 H-tetrazolium-5-carboxanilide)] were acquired from Yuanye Biology (86,386–73-4, 50–04-4, 111,072–31-2). MOPS, db-cAMP, nystatin (NYS), crystal violet (CV), methanol, acetic acid were bought from Macklin (1132–61-1, 16,980–89-5, 1400–61-9, 548–62-9, 67–56-1, 67–19-7). The RPMI-1640 medium for cell culture, was obtained from Servicebio (G4530). The CHROMagar medium was bought from Shanghai Central Bio-Engineering (P001674). The cAMP ELISA kit was obtained from Jianglai Biological (JL51266). Periodic acid Schiff (PAS), PMSF, PBS were gained from Solarbio (G1366, P0100, P1010). Calcofluor white stain (CFW) was bought from Sigma (BCBR3561V). Fetal bovine serum (FBS) was obtained from Gibco (42G5093K). RIPA lysis buffer and penicillin-streptomycin were bought from Beyotime Biology (P10013B, C0222). BCA protein analysis kits were bought from SparkJade (EC0001). Hypoxyprobe^TM^ Green Kit and YC-1 were, respectively, purchased from HPI (HP6-XXX) and Selleck (170,632–47-0). Anti-HIF-1α, HRP conjugated secondary antibody were acquired from Abcam (ab179483, GR3299244-7), while acni-β-actin was obtained from Affinity (AF7018). Electrochemiluminescence kit was bought from ZEN BIO (17,046). IL-17A was bought from PeproTech (061884 D2821). Trizol Reagents was bought from Invitrogen (210,812). SYBR Green Realtime PCR Master Mix, ReverTra Ace qPCR RT Master Mix with gDNA Remover kit were obtained from Toyobo (QPK-201, FSQ-301).

### Anti-biofilm testing

The initial *C. albicans* and *C. glabrata* inoculums were, respectively, adjusted to 1 × 10^6^ cells/mL in RPMI-1640 medium and then incubated with indicated SH, FLU, and SH plus FLU in a 96-well microtiter plate (NEST Biotechnology, Jiangsu, China) for 24 h. The drugs were serially two-fold diluted at 2–512 μg/mL for SH and 8–1024 μg/mL for FLU. The sessile minimum inhibitory concentration (SMIC) was used to examine the susceptibility of the fungi to the drugs by the XTT reduction assay at 492 nm. The endpoint of SMIC_50_ was defined as a 50% decrease of biofilm metabolic activity compared with a drug-free control. The drug interactions were performed by the checkerboard assay. The fractional inhibitory concentration index (FICI) was adopted to survey the interaction between SH and FLU and calculated as FICI = (SMIC_50_-SH in combination/ SMIC_50_-SH alone) + (SMIC_50_-FLU in combination/SMIC_50_-FLU alone). Synergism was defined as FICI ≤ 0.5, indifference as 0.5 < FICI ≤ 4, and antagonism as FICI > 4 [41]. The susceptibility test was also verified by spreading the fungi-containing broth with or without drug treatment onto CHROMagar medium with proper dilutions.

### Biomass quantification

The total biomass of mixed *Candida* biofilms was quantified by CV and the experimental procedures were performed with moderate modification as previously described [42]. The initial inoculum of *C. albicans* (1 × 10^6^ cells/mL) and *C. glabrata* (1 × 10^6^ cells/mL) was co-treated with 8 μg/mL SH and/or 64 μg/mL FLU, and then the supernatant of the mixed fungal culture was pipetted and discarded. Two hundred microliters of methanol were added for 15 min of fixation at room temperature and discarded. The attached cells were stained with 1% CV (m/v) for 5 min and discarded. The wells continued to be washed three times with deionized water. A volume of 33% (v/v) ice acetic acid was incubated for decolorization. The absorbance value was measured at 562 nm with a microplate reader (LabServ K3, Zhennuo Biological Technology, Shanghai, China). The blank control was drug-free with only RPMI-1640.

### Microscopic and macroscopic observation

To analyze the effect of SH and/or FLU on the fungal morphology, a liquid medium and an embedded agar were applied as previously described with fewer modifications [43,44]. There were two conditions used in liquid Spider medium (1% mannitol, 1% nutrient broth, 0.2% K_2_HPO_4_, pH 7.2, m/v). One condition was that *C. albicans* (1 × 10^6^ cells/mL) and *C. glabrata* (1 × 10^6^ cells/mL) were mixed with 8 μg/mL SH and/or 64 μg/mL FLU in the presence/absence of 5 mM db-cAMP in a constant temperature incubator (DHP-9162, Yiheng Scientific Instrument, Shanghai, China) at 37°C for 8 h. The other condition was that the same volumes of the two fungi were co-cultured for 1.5 h without the drug treatments prior to another 4.5 h of co-cultivation with 32 μg/mL SH and/or 256 μg/mL FLU in the presence/absence of 5 mM of db-cAMP. An aliquot of fungal broth was pipetted out on a glass slide and observed with an inverted microscope (OLYMPUS IX51, Tokyo, Japan) at a magnification of ×200. A random sample of 100 cells was selected to record the number of cells containing mycelium. As defined previously, the germ tube of a mycelial cell is at least twice the length of the cell [45]. For embedded agar, a single colony was cultured overnight in YPD liquid medium at 37°C till OD_600_ = 0.1. Two approaches were then adopted in a solid agar medium. In one approach, a volume of 10 mL of YPD agar medium containing 64 μg/mL SH and/or 1 μg/mL FLU was poured into a petri dish and solidified as a lower medium in the presence/absence of 5 mM of db-cAMP. A number of 60–80 of mixed fungal cells were dropped onto the lower medium plate and then coated with a layer of drug-containing YPD agar medium as the upper medium. After solidification, the embedded agar medium was cultivated in a constant temperature incubator at 37°C for 50 h and observed with an upright microscope (OLYMPUS BX51, Tokyo, Japan). The other approach was that the same volume of YPD medium was plated as the lower medium with the similar number of mixed fungal cells and YPD medium as the upper medium for 8 h of drug-free pre-growth prior to the addition of 128 μg/mL SH and/or 2 μg/mL FLU in the presence/absence of 5 mM of db-cAMP for another 40 h of co-cultivation.

### Determination of intracellular cAMP

The intracellular cAMP concentration was measured as reported before with some modifications [46]. After the incubation of *C. albicans* (1 × 10^6^ cells/mL) and *C. glabrata* (1 × 10^6^ cells/mL) with 8 μg/mL SH and/or 64 μg/mL FLU for 8 h, the cells were collected at 825 g of centrifugation at 4°C for 5 min. The supernatant was pipetted and discarded. The pellets were resuspended with a certain amount of 200 μL of PBS, and repeatedly frozen (−20°C, 30 min) and thawed (37°C, 2 min) for three times. The cell lysate was used to detect intracellular cAMP level at 450 nm with a microplate reader (LabServ K3, Zhennuo Biological Technology, Shanghai, China) using a commercial ELISA kit according to the provided protocol.

### Mouse OPC model

All procedures involving animals were approved by the Animal Ethics Committee, Institute of Anhui University of Chinese Medicine (Animal Ethics Number: AHUCM-mouse-2,020,036). Maintenance and treatment of all animals were in compliance with the principles of the Institutional Animal Ethics Committee of the Chinese Center for Disease Control and Prevention and conformed to the Chinese National Guidelines on the Care and Use of Laboratory Animals. The C57BL/6 female mice (20 ± 2 g, 6–7 weeks) were purchased from Jinan Pengyue Laboratory Animal Breeding (Shandong, China). Nine mice were randomly assigned into one group and acclimated for 7–10 days with ad-lib access to tap water and food in a regular 12 h light–dark cycle. The OPC model was established based on several reported literatures with a few adjustments [47,48]. Briefly, before and after infection with dual *C. albicans* and *C. glabrata* co-cultures, a quantity of 225 mg/kg of cortisone acetate was injected intraperitoneally on day −1, +1 and +3. On day 0, the mice were anesthetized intraperitoneally with sodium pentobarbital at 10 mg/mL. The mice are then inoculated by placing a cotton swab saturated with *C. glabrata* ATCC15126 (1 × 10^6^ cells/mL) and *C. albicans* SC5314 (1 × 10^6^ cells/mL) mixed co-cultures sublingually for 75 min. After 24 h of infections, the mice were orally administered with SH at 5.12 mg/kg/d, FLU at 1 mg/kg/d, SH plus FLU at 5.12 + 1 mg/kg/d for four consecutive days. The positive group was given a therapy of NYS at 10 mg/kg/d. The sham mice were provided with an equal volume of sterile deionized water. The weight changes of mice in each group were recorded every day. The mice were euthanized on the sixth day after infections. After execution, the organs and the tongue tissues were excised and stored at −80°C for subsequent experiments.

### Fungal burden

The entire tongue dorsum was wiped with a sterile cotton swab. The swab was then violently vortexed and vibrated for 1 min in sterile PBS. A volume of 100 μL of fungal suspension was smeared on a YPD agar plate containing 1% penicillin-streptomycin. The excised oral tissues and internal organs were totally homogenized by a homogenizer (YJ-0005, Supin Equipment, Jiangsu, China) and resuspended with sterile PBS. After proper dilutions, an aliquot of 40 μL of homogenate suspension was inoculated on YPD agar plate containing 1% penicillin-streptomycin. All of the YPD agar plates were cultivated for 48 h at 37°C for fungal growth.

### PAS staining

After sacrifice, the tongue tissue is excised and fixed in 10% paraformaldehyde, embedded in paraffin, and cut into 5 μm thickness sections. Tissue sections were deparaffinized with xylene and stained with PAS to highlight *Candida* hyphae. The entire periphery of each infected tongue dorsum is examined by a light microscope at a magnification of ×200 based on the presence and degree of adhered yeast cells and the degree of penetration of the epithelium by infiltrating hyphae [49].

### Cell cultivation

The esophageal carcinoma cell EC109 was obtained from the Institute of Basic Medicine, Chinese Academy of Medical Sciences (Beijing, China). The cells were cultured in RPMI-1640 medium containing 10% FBS and 1% penicillin-streptomycin at 37°C in a 5% CO_2_ incubator. A quantity of 1 × 10^5^ cells/mL of EC109 were co-incubated with the mixed fungal cultures of *C. albicans* SC5314 and *C. glabrata* ATCC15126 both at the same inoculum of 1 × 10^6^ cells/mL in the presence of 16 μg/mL SH and/or 8 μg/mL FLU with or without the treatment of YC-1 at 20 μM for 24 h and IL-17A at 50 ng/mL for 24 h.

### Hypoxia staining

The Hypoxyprobe^TM^ Green Kit has been used for immunochemical detection of cell and tissue hypoxia through immunofluorescence in several studies [50,51]. For tissue staining, according to the kit instructions, each mouse was administered intragastrically with 60 mg/kg of Hypoxyprobe^TM^ solution 25 min before sacrifice. Once euthanized, the tongue was wholly removed, embedded in Optimal Cutting Temperature (OCT, REF4583, Sakura, USA), sectioned into 10 µm thickness using a cryostat, fixed in 4% paraformaldehyde for 15 min, and blocked with PBS containing 0.5% Triton X-100 and 10% goat serum at room temperature for 1 h. After aspiration, the sections were completely immersed and incubated with FITC-labeled monoclonal antibody-1 (1:100 dilution) overnight at 4°C. After being washed with sterile PBS every 5 min for three times, the sections were coated with CFW (approximately 20 μL) diluted by 10% (m/v) potassium hydroxide (1:1) for 1 min. The sections were then washed three times with sterile PBS for 5 min each and observed with an inverted fluorescence microscope (DMi8, Leica, Wetzlar, Germany) at ×200. For cell staining, the treated cells were co-incubated with Hypoxyprobe^TM−1^ for 2 h at 37°C. After washing with PBS, the sections were fixed in 4% paraformaldehyde for 15 min, hyalinized in 0.5% Triton X‐100 for 15 min, blocked in 10% goat serum for 1 h, and incubated with FITC-labeled monoclonal antibody-1 (1:100 dilution) overnight at 4°C. After washing with PBS again, the sections were observed with an inverted fluorescence microscope (DMi8, Leica, Wetzlar, Germany) at ×200.

### Flow cytometry

The flow cytometry was performed according to the procedures reported previously [52]. Briefly, an amount of 1 × 10^6^ cells/mL of EC109 was firstly infected by a volume of *C. albicans* SC5314 and *C. glabrata* ATCC15126 dual co-cultures (both at 1 × 10^3^ cells/mL) in the presence of 8 μg/mL SH and/or 0.125 μg/mL FLU at 37°C for 4 h. Secondly, the cells were incubated with Hypoxyprobe^TM^ at 37°C 5% CO_2_ for 2 h and FITC-labeled monoclonal antibody-1 (1:100 dilution) at 37°C for 2 h. After twice washing with sterile PBS, the labeled cells were loaded in a BD FACSCelesta^TM^ flow cytometer (Franklin Lake, NJ, USA). The data were processed using BD FACSDiva 9.0 software at an excitation of 488 nm and an emission of 525 nm. A quantity of approximately 10,000 cells were acquired for the analysis.

### Immunoblot

The treated EC109 cells were lysed using RIPA lysis buffer containing 1% PMSF (final concentration = 1 mmol/L). The total protein was quantified using BCA protein assay kit according to the manufacturer’s instructions. The proteins were separated by a SDS-PAGE device (DYCZ-24 DN, Liuyi, Beijing, China) and transferred to polyvinylidene difluoride (PVDF) membranes (DYCZ-40 D, Liuyi, Beijing, China). The membranes were blocked with 5% nonfat milk for 1 h at room temperature, sequentially incubated with primary antibodies against HIF-1α (1:5000 dilution), β-actin (1:5000 dilution) overnight at 4°C and a HRP conjugated secondary antibody (1:5000 dilution) for 1 h at room temperature. After several washings with TBST, the bands were developed in a Tanon 5200 device (Shanghai, China) and processed by an electrochemiluminescence kit using ImageJ 1.46 r for quantification.

### qRT-PCR

After the scheduled treatment, the total RNA was isolated with Trizol reagent from the tissue samples and EC109 cells in line with the manufacturer’s instructions. The quantity and purity of the extracted RNA were monitored by a DeNovix DS-11 Spectrophotometer (Wilmington, DE, USA). An approximate quantity of 0.1 μg of extracted RNA was loaded and reverse-transcribed into complementary cDNA using a ReverTra Ace qPCR RT Master Mix with gDNA Remover kit according to the manufacturer’s instructions. The cDNA was then diluted 10-fold prior to qRT-PCR. The primers (Table 1) were synthesized by Sangon Biotech (Shanghai, China). The PCR mixture (25 μL in total) was composed of 12.5 µL of SYBR Green Realtime PCR Master Mix, 1 µL PCR Forward Primer, 1 µL PCR Reverse Primer, 0.5 µL cDNA, and 10 µl ddH_2_O. The PCR was performed on an ABI7500 fluorescent quantitative PCR system (Applied Biosystem). The procedures for qRT-PCR are as follows: pre-denaturation at 95°C for 60 s, and then 40 cycles of 95°C for 15 s, 50°C for 15 s, and 72°C for 45 s. All the data were normalized to the housekeeping gene β-actin as the internal reference. The relative expression of the target genes was calculated using the 2^−ΔΔCt^ method [53].

**Table 1.** Primers for qRT-PCR

Primers	Sequence (5’ to 3’)
***β-actin-F***	ACCGAAGCTCCAATGAATCC
***β-actin-R***	CCGGTGGTTCTACCAGAAGAG
***hif-1α-F***	GTCGGACAGCCTCACCAAACAGAGC
***hif-1α-R***	GTTAACTTGATCCAAAGCTCTGAG
***il23-F***	AGTGGAAGTGGGCAGAGATTC
***il23-R***	CAGCAGCAACAGCAGCATTAC
***il17a-F***	AAAGCTCAGCGTGTCCAAA
***il17a-R***	GCGCCAAGGGAGTTAAAGAC
***il17ra-F***	CTAAACTGCACGGTCAAGAAT
***il17ra-R***	ATGAACCAGTACACCCAC

### Statistical analysis

The data were expressed as mean ± standard deviation (SD) of at least three repeated biological replicates. Data analysis was processed by the SPSS 23.0 statistical software package (SPSS, IL, USA) with one-way analysis of variance (ANOVA), followed by the LSD or Welch’s method. The number of fungal cells (Log_10_CFU/mL) isolated from the tongue of infected mice was analyzed by using Student’s *t*-test. The fungal burdens of the five organs (liver, spleen, kidney, stomach, and colon, each from seven mice) are analyzed by the Kruskal–Wallis test with Dunn’s posttest for multiple comparisons. P < 0.05 was considered significant.

## Results

### *SH is synergistic with FLU to inhibit cAMP mediated filamentation in dual* Candida *biofilms*

**Figure 1.**
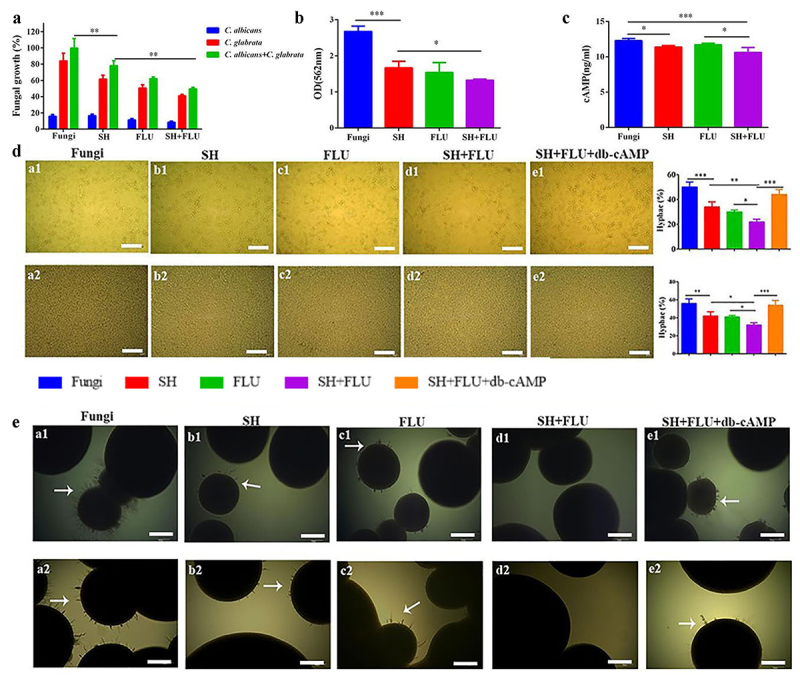
SH and FLU are synergistic against the dual *C. albicans* and *C. glabrata* biofilms through inhibiting filamentation in vitro. **A**. The drugs inhibit the single and dual fungal growth in the mixed biofilms by cell counting. **B**. The drugs reduce the biomass of the mixed fungal biofilms by CV staining. **C**. The drugs inhibit the metabolism of the mixed fungal biofilms by intracellular cAMP determination. The final concentrations of SH and FLU are at 8 and 64 μg/mL in **A-C. D**. Filamentation of the mixed fungal biofilms can be abolished by (**a1-e1**) 8 μg/mL SH and/or 64 μg/mL FLU after 8 h of co-cultivation and (**a2-e2**) 32 μg/mL SH and/or 256 μg/mL FLU for 4.5 h of co-cultivation after 1.5 h of drug-free pre-growth, and compensated by db-cAMP at 5 mM in a liquid Spider medium. The fungal cell with germ tube is defined as that described in the **Materials and Methods**. The histogram is the analysis of 100 fungal cells in three different occasions. Scale bar: 50 μm. **E**. Mycelium of the mixed fungi can be abrogated by (**a1-e1**) 64 μg/mL SH and/or 1 μg/mL FLU after 50 h of co-growth and (**a2-e2**) 128 μg/mL SH and/or 2 μg/mL FLU for 40 h of co-cultivation after 8 h of drug-free pre-incubation, and compensated by db-cAMP at 5 mM in a solid embedded agar condition. Scale bar: 100 μm. * p < 0.05, ** p < 0.01, *** p < 0.001. Fungi: *C. albicans* SC5314 and *C. glabrata* ATCC15126.

Although the antifungal susceptibility of SH to *C. albicans* planktonic cells has been performed [38], its effect on *C. albicans* and *C. glabrata* dual biofilms is unknown up to now. As shown, the SMIC_50_ of SH and FLU against *C. albicans* SC5314, Z215 and *C. glabrata* ATCC15126, ATCC28226 single biofilms are varying between 32 and 64 and 8->1024 μg/mL (Table 2). Impressively, SH alone has favorable activity against the four dual *Candida* biofilms (SMIC_50_ = 128 μg/mL), compared with the high resistance of FLU (SMIC_50_ > 1024 μg/mL). After combination, SH and FLU present synergism at 8 and 64 μg/mL by XTT assay (FICI <0.125, Table 2). To avoid experimental inconsistencies caused by the diverse metabolic activities of *C. albicans* and *C. glabrata* [54], plate counting is used to further determine the reliability of SMIC_50_ in the four dual *Candida* biofilms. As presented, SH causes a growth decrease of 83.5% and 38.5% of *C. albicans* SC5314 and *C. glabrata* ATCC15126 in their mixed biofilms, compared with 88.6% and 49.5% caused by FLU (Figure 1a). Importantly, an approximate of 50.5% of the mixed fungal growth was inhibited by the combination of SH and FLU, compared with 22% by SH and 38.1% by FLU in *C. albicans* SC5314 and *C. glabrata* ATCC15126 dual biofilms (Figure 1a). What is more, the combined drugs can make a 39.3–47.3% decrease of fungal growth in the other three dual biofilms at 8 and 64 μg/mL for SH and FLU (Figure S1A). The CV assay and the intracellular cAMP determination also demonstrate the synergism of SH and FLU (Figure 1b-C and S1B-C). Due to the indispensable role of hypha in dual *C. albicans* and *C. glabrata* biofilms [13], the effects of SH and/or FLU on filamentation are examined in the presence of db-cAMP, which has nice lipid solubility and can promote hyphal growth [55]. Whether in liquid or solid culture conditions, *C. albicans* can develop typical hyphae, while *C. glabrata* is unable to form filaments (Figure S2). In liquid medium, the single use of SH or FLU can significantly decrease the fungal growth and filament elongation in both co-incubated and pre-incubated conditions. On this basis, the drug combination can further remarkably increase hyphal inhibition in all of the four *C. albicans* and *C. glabrata* co-cultures. The mycelial decrease can be reversed by extraneous db-cAMP at 5 mM (Figure 1d and Figure S3). To our surprise, we fail to observe mycelium around the colonies on the surface of solid agar when *C. albicans* is co-grown with *C. glabrata* in the four strains (data not shown). Instead, we test the effects of SH and/or FLU on filamentation in an embedded solid agar condition. Consistent with the results in liquid mediums, no hypha can be visually found after the dual use of SH and FLU in comparison with their single administration in both co-incubated and pre-incubated conditions. Similarly, exogenous db-cAMP counteracts the effect of SH plus FLU (Figure 1e and S4).

**Table 2.** Antifungal activities of SH and FLU against *Candida albicans* and *Candida glabrata* single/dual biofilms

Drugs	SMIC_50_ (μg/mL)							
	A	B	C	D	A + C	A + D	B + C	B + D
**SH**	64	64	32	32	128	128	128	128
**FLU**	8	512	>1024	>1024	>1024	>1024	>1024	>1024
**SH+FLU**	**–**	**–**	**–**	**–**	8/64	8/64	8/64	8/64
**FICI**	**–**	**–**	**–**	**–**	<0.125	<0.125	<0.125	<0.125
**Interpretation**	**–**	**–**	**–**	**–**	Synergism	Synergism	Synergism	Synergism

A: *C. albicans* SC5314; B: *C. albicans* Z215; C: *C. glabrata* ATCC15126; D: *C. glabrata* ATCC28226.

### *SH increases FLU therapy in a dual* Candida *biofilms induced OPC mouse model via inhibiting filamentation*

**Figure 2.**
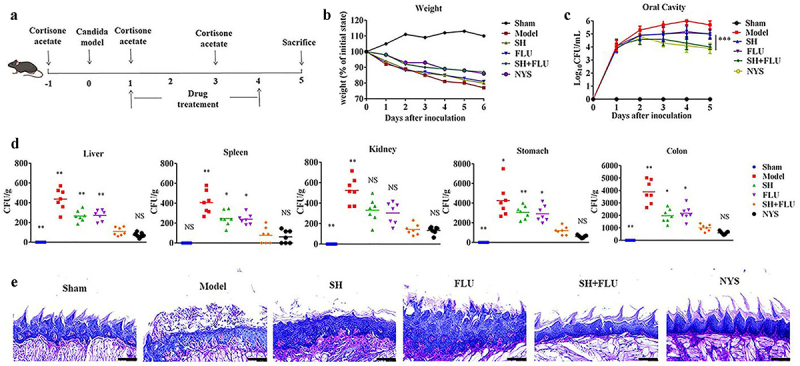
SH and/or FLU can protect the mice from OPC caused by *C. albicans* SC5314 and *C. glabrata* ATCC15126. **A**. Scheme of experimental design. **B**. Body weight assessment of mice. **C**. Changes of oral fungal burden (n = 3). **D**. Fungal capacities of liver, spleen, kidney, stomach and colon (n = 7). **E**. Examination of fungal colonization and penetration of infected mucosal tongue tissues by periodic acid Schiff (PAS) staining. Scale bar: 50 μm. * p < 0.05, ** p < 0.01, *** p < 0.001.

*C. albicans* and *C. glabrata* are readily co-isolated in oral cavities and deemed as culprits in OPC development [13]. In this study, an acute OPC mouse model induced by co-infection of *C. albicans* and *C. glabrata* is established to evaluate the mono- and dual therapy of SH and FLU (Figure 2a). At the end of the experiment, the mouse weight loses approximately 10% after the co-treatment of SH and FLU compared with nearly 20% post-SH/FLU solo therapy (Figure 2b). The fungal burden post the combined therapy is comparable to that after NYS treatment and superior to that by single-drug application in oral cavity (Figure 2c). Except kidney, SH plus FLU can markedly reduce the fungal capacity in liver, spleen, stomach, and colon compared to their solo use. Also, the dual-drug therapy inhibits internal fungal growth as effectively as NYS in the five organs (Figure 2d). The etiological results are further verified by pathological examinations. Clearly, the overgrown fungal filaments cause the impairment of lingual dorsum papilla and oral mucosal epithelium in the model group. SH or FLU single employment is unable to reconstitute papilla structure and remove the colonized fungi. However, the drug combined therapy can effectively get rid of all forms of fungal cells, recover papilla, and oral mucosal epithelium, accompanied by reduced inflammation (Figure 2e). These results demonstrate that SH in combination with FLU is capable of reducing fungal capacity and alleviate oral inflammation by inhibiting filamentation in the dual *Candida* biofilms induced OPC mouse model.

### *SH plus FLU regulates HIF-1α/IL-17 axis to ameliorate dual* Candida *biofilms induced oral infections*

**Figure 3.**
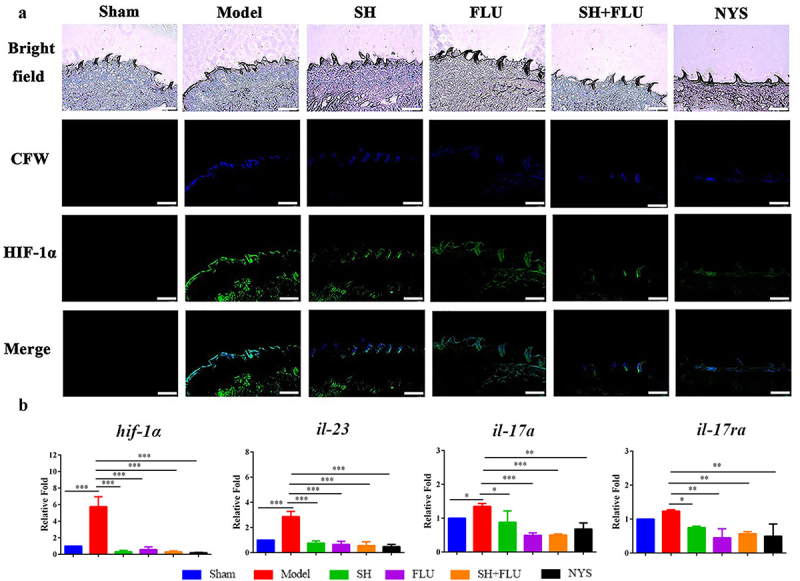
SH and/or FLU affect HIF-1α/IL-17 axis in the OPC model induced by *C. albicans* SC5314 and *C. glabrata* ATCC15126. **A**. SH and/or FLU depress the dual fungal biofilms (stained by CFW, blue) associated hypoxic microenvironment (stained by Hypoxyprobe^TM^, green) in the oral mucosal surface of OPC mice. Scale bar: 50 μm. **B**. SH and/or FLU downregulate the mRNA expressions of *hif-1α, il-23, il-17a* and *il-17ra* mRNA in vivo. * p < 0.05, ** p < 0.01, *** p < 0.001.

**Figure 4.**
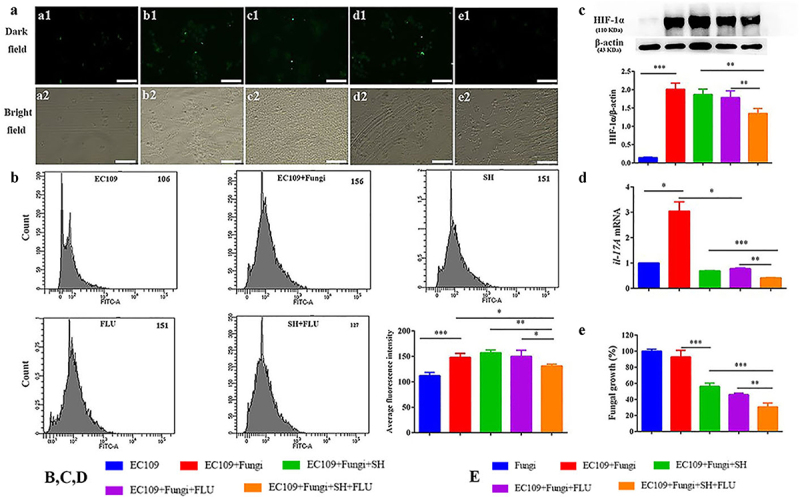
SH and/or FLU inhibit the mixed fungal biofilms via the regulation of HIF-1α/IL-17 axis in EC109. **A**. Representative fluorescent images of EC109 cells in the dark (a1-e1) and bright (a2-e2) fields show an inhibition of the drug combination on fungal colonization induced hypoxia stained by Hypoxyprobe^TM−1^. **a1-a2**, EC109; **b1-b2**, EC109+ Fungi; **c1-c2**, EC109+ Fungi+SH; **d1-d2**, EC109+ Fungi+FLU; **e1-e2**, EC109+ Fungi+SH+FLU; Scale bar: 50 μm. **B**. Representative flow cytometry profiles and analysis of EC109 cells labeled by FITC-Hypoxyprobe^TM−1^ reveal a decrease of hypoxic EC109 after drug combination. The fluorescent intensity of each representative profile is marked top right. **C**. Representative Western blot bands and quantitative analysis exhibit a reduction of HIF-1α protein by SH and/or FLU in EC109. **D**. SH and/or FLU can make a downregulation of *il-17a* mRNA expression in EC109. **E**. SH and/or FLU can cause a decline of fungal growth after the incubation with EC109. The experimental methods of **C-E** are the same as those described in **A**. * p < 0.05, ** p < 0.01, *** p < 0.001. Fungi: *C. albicans* SC5314 and *C. glabrata* ATCC15126.

Hypoxia is a common feature of inflammation, and both fungal and bacterial biofilms can form hypoxic microenvironments [21,56]. In our OPC mouse model, two fluorescent dyes, i.e. CFW (blue) and Hypoxyprobe (green), are used to label fungal cell walls and hypoxic niches. After dual fungal infections, it is evident that the hypoxic oral mucosal surface is colonized by the dual fungal cells. Furthermore, the damaged dorsum papilla facilitates the generation of subcutaneous hypoxia (Figure 3a and S5). The fluorescent co-localization of CFW and Hypoxyprobe demonstrates that the formation of *C. albicans* and *C. glabrata* dual biofilms contributes to the development of low-oxygen. Relative to the treatment of SH or FLU, the concomitant medication of SH and FLU prominently shrinks the anoxic area and subdues the dual fungal colonization in a simultaneous way (Figure 3a). The qRT-PCR results show that the mRNA expressions of *hif-1α, il-17a,* and *il-23* are all significantly upregulated in the model group. Compared with mice free of drug treatment, the mono- and dual-therapies of SH and FLU can remarkably downregulate the mRNA expressions of *hif-1α, il-17a, il-17ra,* and *il-23*. The effectiveness of the drug combination does not overwhelm that of the drug mono-use but is close to that of NYS (Figure 3b). We then use EC109, an esophageal carcinoma cell line, which has IL-17A receptor [57], to further investigate the therapeutic mechanism of SH plus FLU in the treatment of OPC. Similar to the results in the animal experiments, compared with the single use, SH plus FLU can significantly restrict hypoxic microenvironment (Figure 4a), reduce the hypoxia-induced fluorescent intensity (Figure 4b), inhibit the level of HIF-1α protein (Figure 4c), downregulate the expression of *il-17a* mRNA (Figure 4d), and decrease the viable fungal cells (Figure 4e), when EC109 is co-incubated with the fungal co-cultures.

### *SH plus FLU restrains fungal growth to indirectly affect HIF-1α/IL-17 axis in dual* Candida *biofilms induced oral infections*

**Figure 5.**
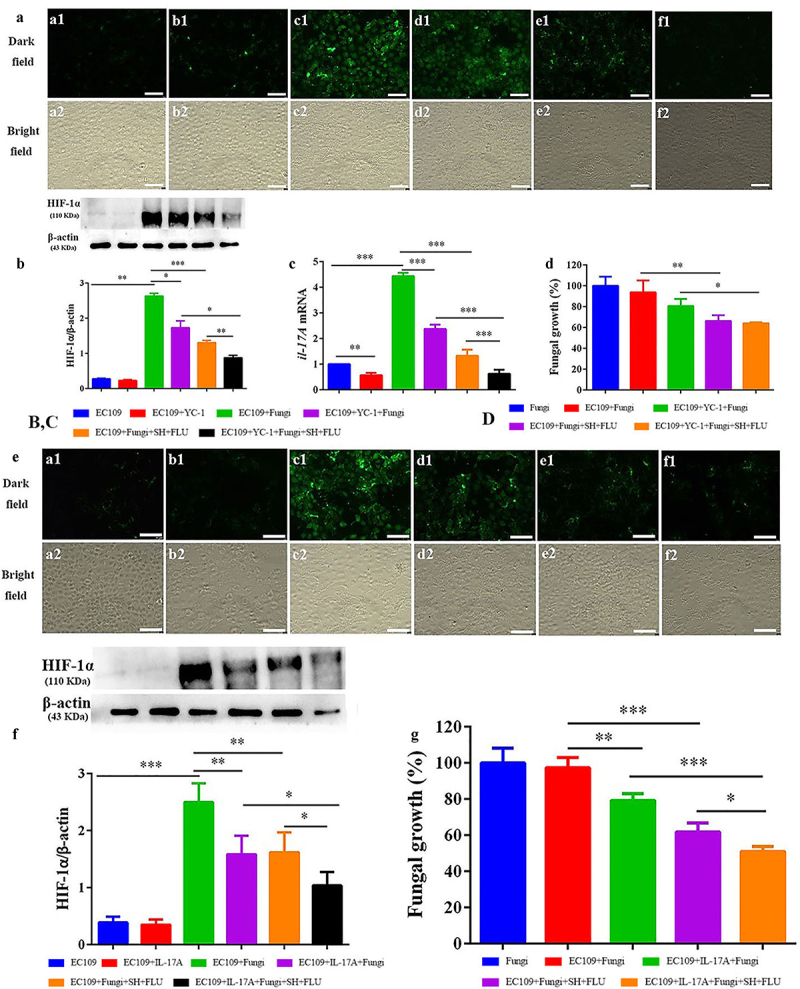
Inhibition of the mixed fungal co-cultures by SH plus FLU is indirectly associated with the regulation of the HIF-1α/IL-17 axis in EC109 cells treated with YC-1 or IL-17A. **A, E**. Representative fluorescent images of EC109 cells stained by Hypoxyprobe^TM−1^ in the dark (a1-f1) and bright (a2-f2) fields show a synergistic inhibition of YC-1 (HIF-1α inhibitor)/IL-17A and the drug combination on fungal colonization induced hypoxia. **a1-a2**, EC109; **b1-b2**, EC109+ YC-1/IL-17A; **c1-c2**, EC109+ Fungi; **d1-d2**, EC109+ YC-1/IL-17A +Fungi; **e1-e2**, EC109+ Fungi+SH+FLU; **f1-f2**, EC109+ YC-1/IL-17A +Fungi+SH+FLU. Scale bar: 50 μm. **B, F**. Representative Western blot bands and quantitative analysis exhibit a synergistic inhibition of YC-1/IL-17A and SH plus FLU on HIF-1α protein in EC109 cells. **C**. YC-1 promotes the inhibitory effect of SH plus FLU on *il-17a* mRNA expression in EC109 cells. **D, G**. The mixed fungal growth can be restrained with the treatments of YC-1/IL-17A and SH plus FLU. * p < 0.05, ** p < 0.01, *** p < 0.001. Fungi: *C. albicans* SC5314 and *C. glabrata* ATCC15126.

The addition of YC-1, a specific inhibitor of HIF-1α [58], has negligible impact on the hypoxic microenvironment in EC109 (Figure 5a). Since the normal HIF-1α level is very low (Figures 4C, 5b, 5f, and S6A), the inhibition of YC-1 on HIF-1α is visually faint (Figure 5b). However, YC-1 can significantly downregulate the expression of *il-17a* mRNA (Figure 5c). Impressively, YC-1 can strongly promote the inhibition of the combined effect of SH and FLU on HIF-1α level and *il-17a* mRNA expression compared with the single therapy of YC-1 or the drug combination (Figure 5b and 5C), although this effect is insignificant in fungal growth (Figure 5d). In line with the YC-1 results, the exogenous IL-17A can also strikingly enhance the synergy of SH plus FLU to cripple hypoxia, HIF-1α level, as well as the fungal growth compared with the individual use of IL-17A or the drug combination (Figure 5e-G). To uncover the mode of action of the drugs, we find that SH and FLU alone and in combination have no evident impacts on HIF-1α level in EC109 (Figure S6A) and YC-1 and IL-17A alone are also unable to control fungal growth (Figure S6B). Considering the antifungal activities of SH and FLU (Figure 1), we speculate that SH plus FLU exerts an indirect impact on HIF-1α/IL-17 axis via inhibiting fungal proliferation. To decipher the role of EC109 in the antifungal function of the drug combination, the anti-biofilm potential of SH and FLU is surveyed in the absence of EC109 at 4 and 24 h. Interestingly, the combined drugs display stronger inhibition on the mixed fungal growth in the presence of EC109 than in the absence of EC109 (30.6% versus 53.4%, Figure 4e and Figure S6C). Furthermore, the supernatant of EC109 stimulated by the mixed fungi could significantly decrease the fungal growth compared with that of unstimulated EC109 (Figure S6D). These results demonstrate that EC109 might secrete unidentified actives to assist the combined drugs to combat against the dual *Candida* biofilms. We also note that SH plus FLU can cause a lower decrease in fungal growth (~72%) in the presence of unstimulated supernatant of EC109 (Figure 4d) than that (53.4%) in the in vitro test after 4 h of incubation (Figure S6C). It is assumed that the different components contained in the medium used in the in vitro susceptibility test and the cell culture might be responsible for the discrepancy of fungal inhibition by the drug combination.

## Discussion

Traditional activities are the first choice for the treatment of mixed fungal infections. The biofilm phenotype of *C. albicans* confers a high resistance to these commonly used drugs. More severely, *C. glabrata* is intrinsically resistant to azole and can readily acquire drug resistance during prolonged azole administration, either in vitro or in vivo [59]. Combinatorial use of conventional antifungals and plant-derived compounds with antifungal activity is a feasible strategy to overcome the drug resistance. Herein, we show that SH is synergistic with FLU to inhibit *C. albicans* and *C. glabrata* dual biofilms. One of the underlying mechanisms of the combinatorial approach is the synergistic multi-target effects [60]. In fact, SH and FLU might have diverse anti-*Candida* targets. FLU can disrupt the synthesis of ergosterol in fungal cell membranes by inhibiting cytochrome P450-dependent enzyme lanosterol 14-α-demethylase involved in the conversion of lanosterol to ergosterol [61]. Our preliminary efforts show that the antifungal mechanism of SH against *C. albicans* is possibly related with the transportation and synthesis of β-glucan [38,62]. This study reveals that the major mode of action of SH plus FLU is the direct inhibition on the fungal growth instead of on the HIF-1α/IL-17 axis in oral infections.

Filamentation is required for the formation of *C. albicans* and *C. glabrata* dual biofilms. Due to the uncommon occurrence of pseudohyphae and hyphae in *C. glabrata* [63], yeast-to-hypha transition in the dual *Candida* biofilms is up to *C. albicans* to a large extent, and the hyphal growth of *C. albicans* is mainly regulated by cAMP. By co-staining with Hypoxyprobe^TM^ and CFW, we notice that the fungal dual biofilms induce hypoxic microenvironment and cause the damages of tongue stratum corneum and papilla in a mouse OPC model. This suggests a close relationship between hypha-induced hypoxia and oral inflammation. Indeed, HIF-1α can be readily activated by pathogens including bacteria, viruses, protozoa, as well as fungi [21,64–66]. Nadine and the colleagues reported that *C. albicans* can induce a strong HIF-1α signal in keratinocytes, dermal capillaries, neutrophils, dermal lymphocytes and macrophages, and subcorneal neutrophils [67]. For the first time, we correlate the hypha with HIF-1α in the OPC progression induced by dual *Candida* biofilms. In the cell and animal experiments, our results further show that the combined drug administration can significantly inhibit fungal growth and reduce HIF-1α level as well as the hypoxic niche.

HIF-1α is a key regulator of IL-17 in OPC [30]. However, the correlation between HIF-1α and IL-17 is obscure in oral infections. In this report, the expression of IL-17A mRNA is downregulated when HIF-1α is inhibited, and the HIF-1α protein level is depressed by exogenous IL-17A. These results confirm that there is a close correlation between HIF-1α and IL-17 in oral cavity. Our data also show that the dual fungi exhibit higher susceptibility to the combined drugs in the presence of EC109 than those free of oral cells. Further results demonstrate that there are active compounds that can assist the combined drugs against the mixed fungi in the supernatant secreted by EC109 after fungi stimulation. Actually, IL-17A can strongly induce a group of antimicrobial peptides (AMPs) including BD3 (the ortholog of human BD2), S100A8/9 in oral cavity [25]. These AMPs are regarded as crucial products of oral mucosal epithelia and possess compelling candidacidal activity [2]. These peptides are also an integral part of the innate immune system in the defense of oral mucosal integrity and function [68]. As a result, we suppose that IL-17A might induce the oral cells to secrete AMPs, which can synergistically interact with the combined drugs to combat against the dual *Candida* infections.

Hypoxic microenvironment generated by biofilms is not readily eradicated in vivo. First of all, oxygen deficit can drive fungal resistance to traditional antifungals ^20^. Secondly, anoxia can assist *C. albicans* to evade immune surveillance by cell wall masking of β-glucan, a pathogen associated molecular pattern (PAMP) that could be recognized by dectin-1 on the surface of immune cells [69,70]. However, increasing evidence demonstrates that several drugs can induce exposure of the cell wall β-glucan and activate the innate immune response to invaded *C. albicans* [71,72]. Fortunately, Arnab et al. and our group found that both FLU and SH had the ability of inducing cell wall β-glucan exposure in *C. albicans* [62,73]. Since the major polysaccharide of the cell wall of *C. glabrata* is still β-glucan which can also be exposed by antifungals [74], we infer that the mono- and dual-uses of SH and FLU can induce the unmasking of β-glucan of cell walls in *C. glabrata*. In a previous study by Arnab et al., hypoxia induced β-glucan masking was mediated by cAMP-PKA pathway [70]. In fact, Hyunsook and the colleagues have verified that the fungal CAMP-PKA pathway dictates epithelial cell interactions during OPC [75]. Consequently, cAMP pathway might be involved in hypoxia associated filamentation and β-glucan exposure. Because mice are naïve to *C. albicans*, the first encounter will trigger an innate immune response by producing mainly IL-17 [25,26,28,76], and the innate immunity are exquisitely modulated by HIF-1α [77]. In oral cavity, the ephrin type-A receptor 2 (EphA2) may be the primary sensor of exposed β-glucan [78–80]. Intriguingly, Marc and the colleagues indicated that EphA2 might be indispensable for a maximal IL-17 response in OPC [80]. After ligation between the exposed β-glucan and EphA2, the immune cells (such as neutrophils) are then activated in response to *C. albicans* and *C. glabrata* dual biofilms in OPC. Therefore, the involvement of EphA2 as well as dectin-1 in discerning-exposed β-glucan might be one of the therapeutic mechanisms of SH and/or FLU in OPC. Relevant investigations are under way.

**Figure 6.**
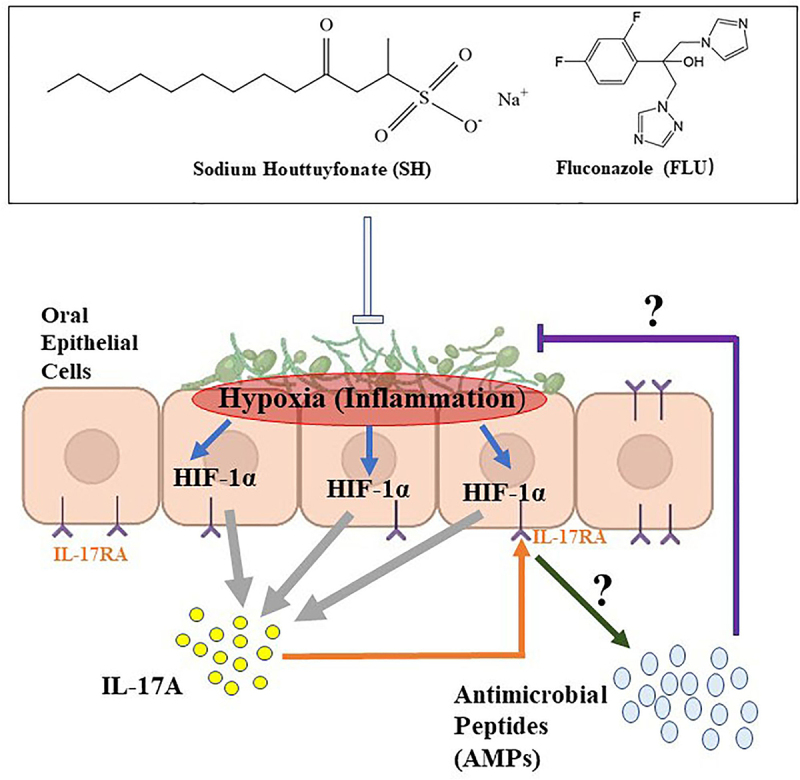
Summarized findings of this work.

Taken together, the main findings can be summarized as follows (Figure 6): when mucosal barrier is damaged, the invasion of fungi (*C. albicans* and *C. glabrata* in this study) is able to form biofilms. The biofilm formation can create a hypoxic microenvironment, trigger activation of HIF-1α/IL-17 axis, and promote OPC. Upon the drug therapy of SH and FLU, the fungal filamentation is inhibited, and the biofilms are disorganized, making the fungal cells oxygenated (decreased HIF-1α protein). As a matter of fact, Caitlin and the colleagues observed that the fungal survival was decreased upon oxygenation of *Aspergillus fumigatus* biofilms [20]. With the removal of hypoxic niche, the oral inflammations are alleviated following the decreased IL-17 level. During this process, the oral mucosal cells might cooperate with the drugs to get rid of the fungi by stimulating AMPs.

## Supplementary Material

Supplemental Material

## Data Availability

The data and materials that support the results or analyses presented in this study are available from the corresponding author on reasonable request.
