# Supplementary material for: Sodium houttuyfonate enhances the mono-therapy of fluconazole on oropharyngeal candidiasis (OPC) through HIF-1α/IL-17 axis by inhibiting cAMP mediated filamentation in Candida albicans-Candida glabrata dual biofilms
Source: Virulence. 2022 Feb 23;13(1):428–43. doi: 10.1080/21505594.2022.2035066 (PMC8890385; doi:10.1080/21505594.2022.2035066)
Supplement: Supplemental Material [file KVIR_A_2035066_SM2189.doc]

**Supporting information for**

Sodium houttuyfonate enhances the mono-therapy of fluconazole on oropharyngeal candidiasis (OPC) through HIF-1α/IL-17 axis by inhibiting cAMP mediated filamentation in *Candida albicans-Candida glabrata* dual biofilms

**Running title:** OPC therapy of SH and FLU

Mengli Chen^1,♀^, Ting Cheng^1,♀^, Chen Xu^1,♀^, Min Pan^1^, Jiadi Wu^4^, Daqiang Wu^1,2,5^, Guiming Yan^1,2^, Tianming Wang^1,3,*^, Changzhong Wang^1,2^, Jing Shao^1,2,*^

^1^Laboratory of Infection and Immunity, College of Integrated Chinese and Western Medicine (College of Life Science), Anhui University of Chinese Medicine, 436 Room, Zhijing Building, 350 Longzihu Road, Xinzhan District, Hefei 230012, Anhui, P. R. China;

^2^Institute of Integrated Traditional Chinese and Western Medicine, Anhui Academy of Chinese Medicine, Zhijing Building, 350 Longzihu Road, Xinzhan District, Hefei 230012, Anhui, P. R. China;

^3^Inflammation and Immune Mediated Diseases Laboratory of Anhui Province, School of Pharmacy, Anhui Medical University, 81 Meishan Road, Hefei 230032, P. R. China

^4^Department of Anatomy, School of Basic Medicine, Huazhong University of Science and Technology

^5^CAS Center for Excellence in Molecular Cell Sciences, Ministry of Education Key Laboratory for Membrane-less Organelles & Cellular Dynamics, Hefei National Laboratory for Physical Sciences at the Microscale, School of Life Sciences, Division of Life Sciences and Medicine, University of Science and Technology of China, 230027 Hefei, P.R. China

^♀^ These authors contribute equally to this paper.

^*^ Corresponding authors:

Dr. Tianming Wang, E-mail: wtm1818@163.com;

Dr. Jing Shao, E-mail: ustcnjnusjtu@126.com;

Tel/Fax: +86-551-6812-9457.


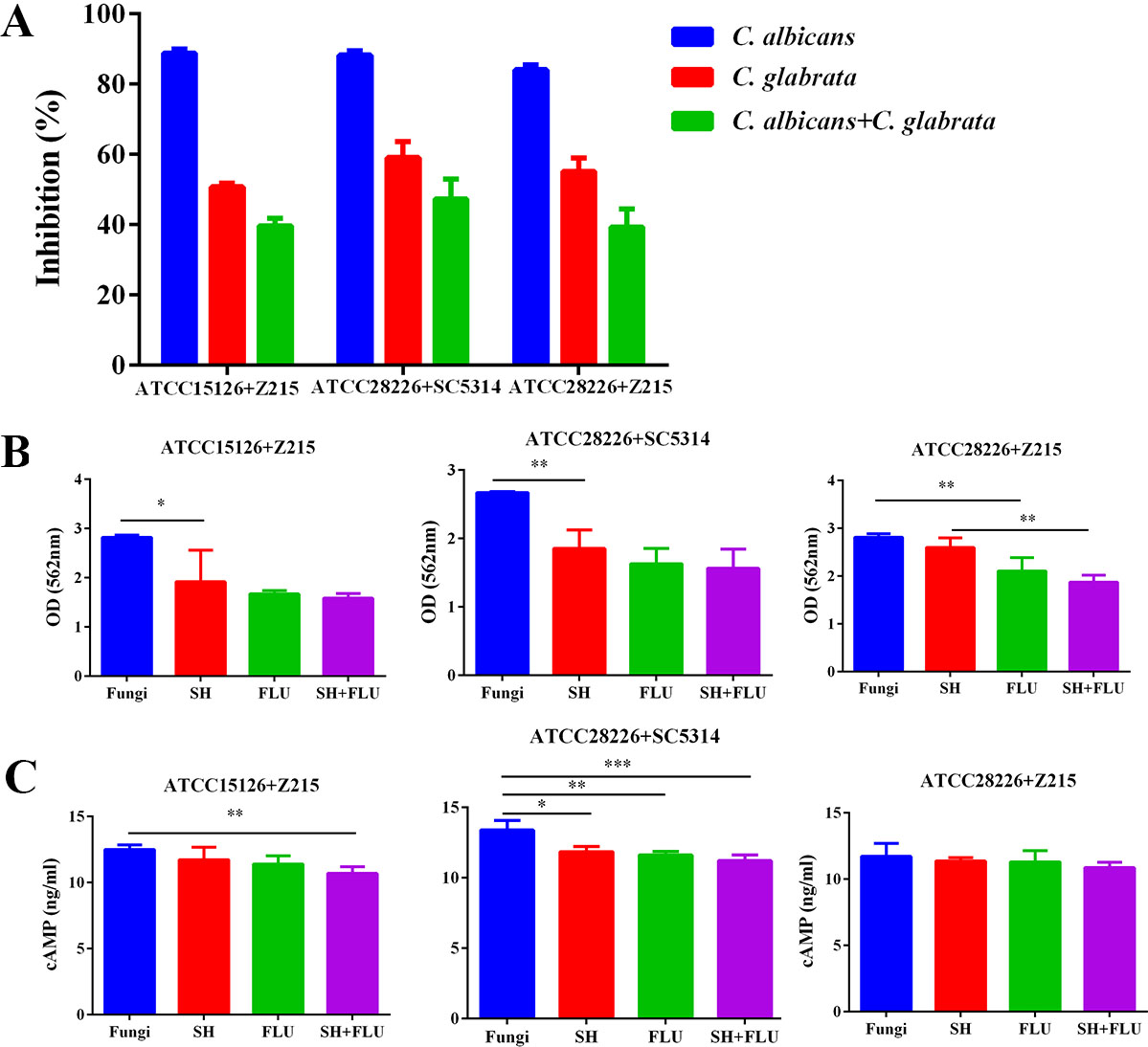


**Figure S1.** In vitro effects of SH and/or FLU on the dual biofilms of *C. albicans* SC5314 and *C. glabrata* ATCC 28226, *C. albicans* Z215 and *C. glabrata* ATCC 15126, *C. albicans* Z215 and *C. glabrata* ATCC 28226. **A.** The drugs restrict the single and mixed growth of the fungi in the dual *Candida* biofilms after the treatment of 8 μg/mL SH in combination with 64 μg/mL FLU by cell counting. **B.** The drugs reduce the biomass of the mixed biofilms at 8 μg/mL SH, 64 μg/mL FLU, and 8+64 μg/mL SH+FLU by CV staining. **C.** The drugs inhibit the intracellular cAMP content of the mixed biofilms at 8 μg/mL SH, 64 μg/mL FLU, and 8+64 μg/mL SH+FLU by a commercial ELISA kit.* p<0.05, ** p<0.01, *** p<0.001.


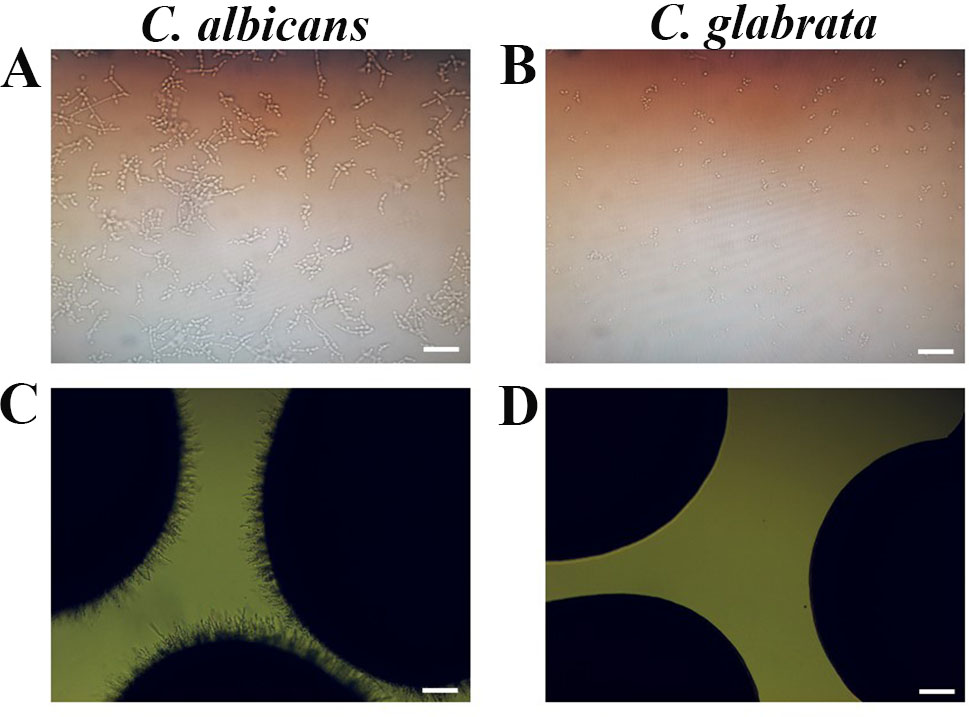


**Figure S2.** (**A-B**) Inverted microscopic observation of *C. albicans* and *C. glabrata* in spider liquid medium after 8 h of incubation. Scale bar: 50 μm**.** (**C-D)** Upright microscopic observation of *C. albicans* and *C. glabrata* in solid embedded agar medium after 50 h of incubation. Scale bar: 100 μm.


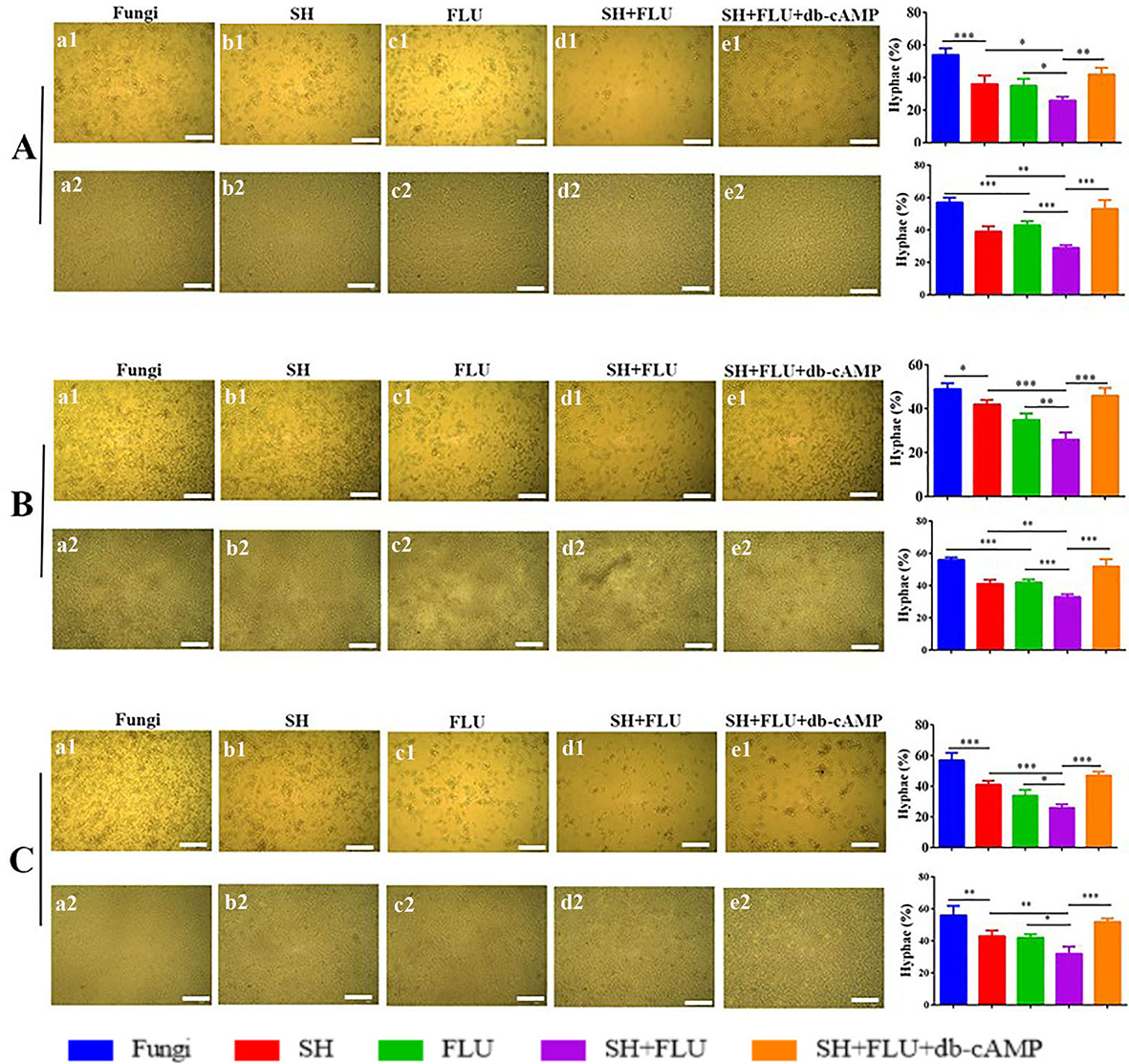


**Figure S3.** Filamentation of the mixed biofilms of (**A)** *C. albicans* Z215 and *C. glabrata* ATCC 15126, (**B)** *C. albicans* SC5314 and *C. glabrata* ATCC 28226, (**C)** *C. albicans* Z215 and *C. glabrata* ATCC 28226 can be abolished by SH and/or FLU, and compensated by db-cAMP in the presence of SH plus FLU in a liquid Spider medium. Scale bar: 50 μm. The experimental methods are the same as those described in Figure 1D. * p<0.05, ** p<0.01, *** p<0.001.


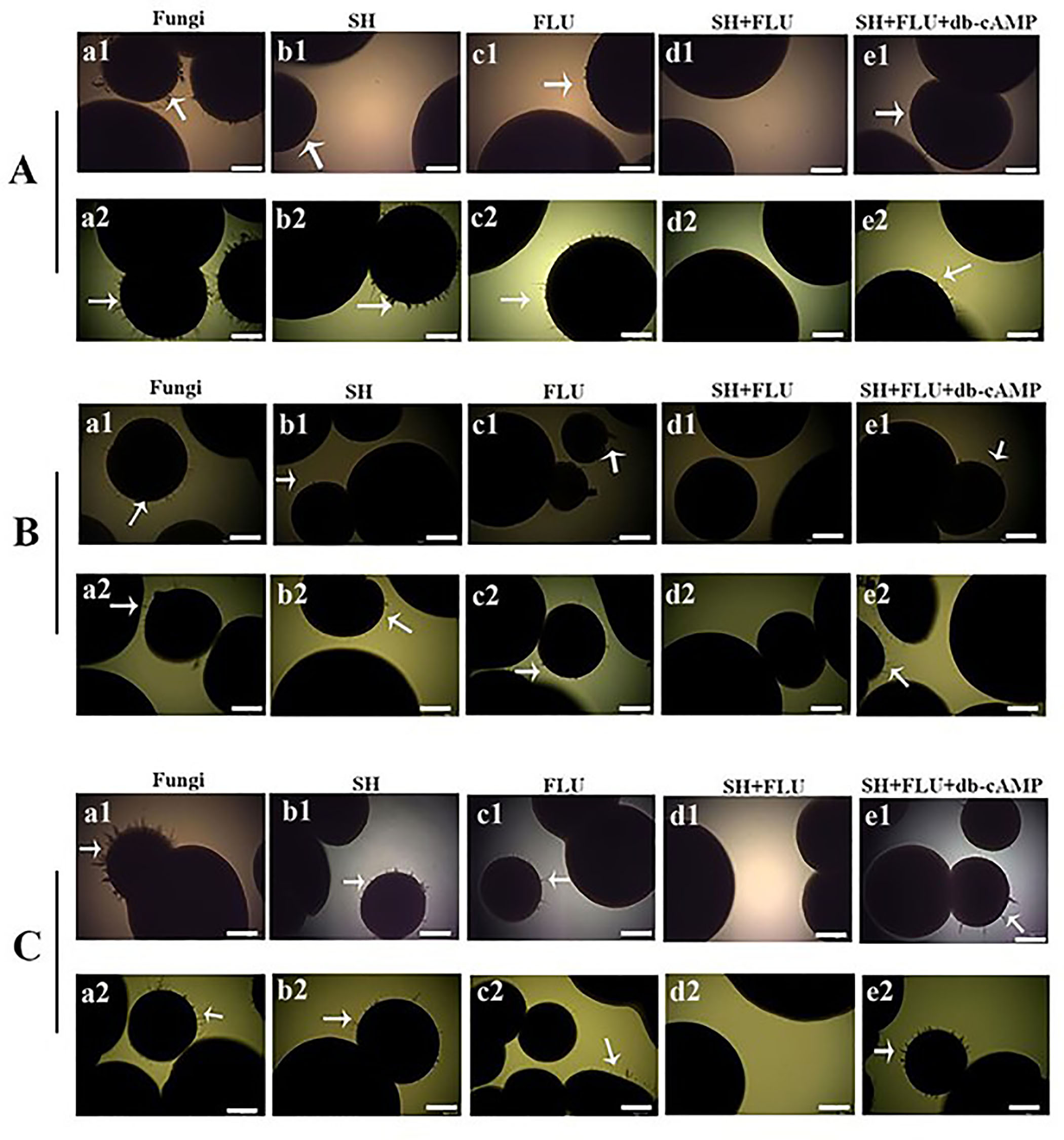


**Figure S4.** Mycelium of the mixed biofilms (**A)** *C. albicans* Z215 and *C. glabrata* ATCC 15126, (**B)** *C. albicans* SC5314 and *C. glabrata* ATCC 28226, (**C)** *C. albicans* Z215 and *C. glabrata* ATCC 28226 can be abrogated by SH and/or FLU, and compensated by db-cAMP in the presence of SH plus FLU in a solid embedded agar condition. In *C. albicans* Z215 and *C. glabrata* ATCC 15126, the drugs is incubated with the dual biofilms for 51 h in a1-e1, and with 8 h of pre-grown mixed fungal biofilms for another 42 h in a2-e2. In *C. albicans* SC5314 and *C. glabrata* ATCC 28226, the drugs is incubated with the dual biofilms for 52 h in a1-e1, and with 8 h of pre-grown mixed fungal biofilms for another 43 h in a2-e2. In *C. albicans* Z215 and *C. glabrata* ATCC 28226, the drugs is incubated with the dual biofilms for 47 h in a1-e1, and with 8 h of pre-grown mixed fungal biofilms for another 48 h in a2-e2. Scale bar: 100μm. Other experimental methods are the same as those described in Figure 1E.


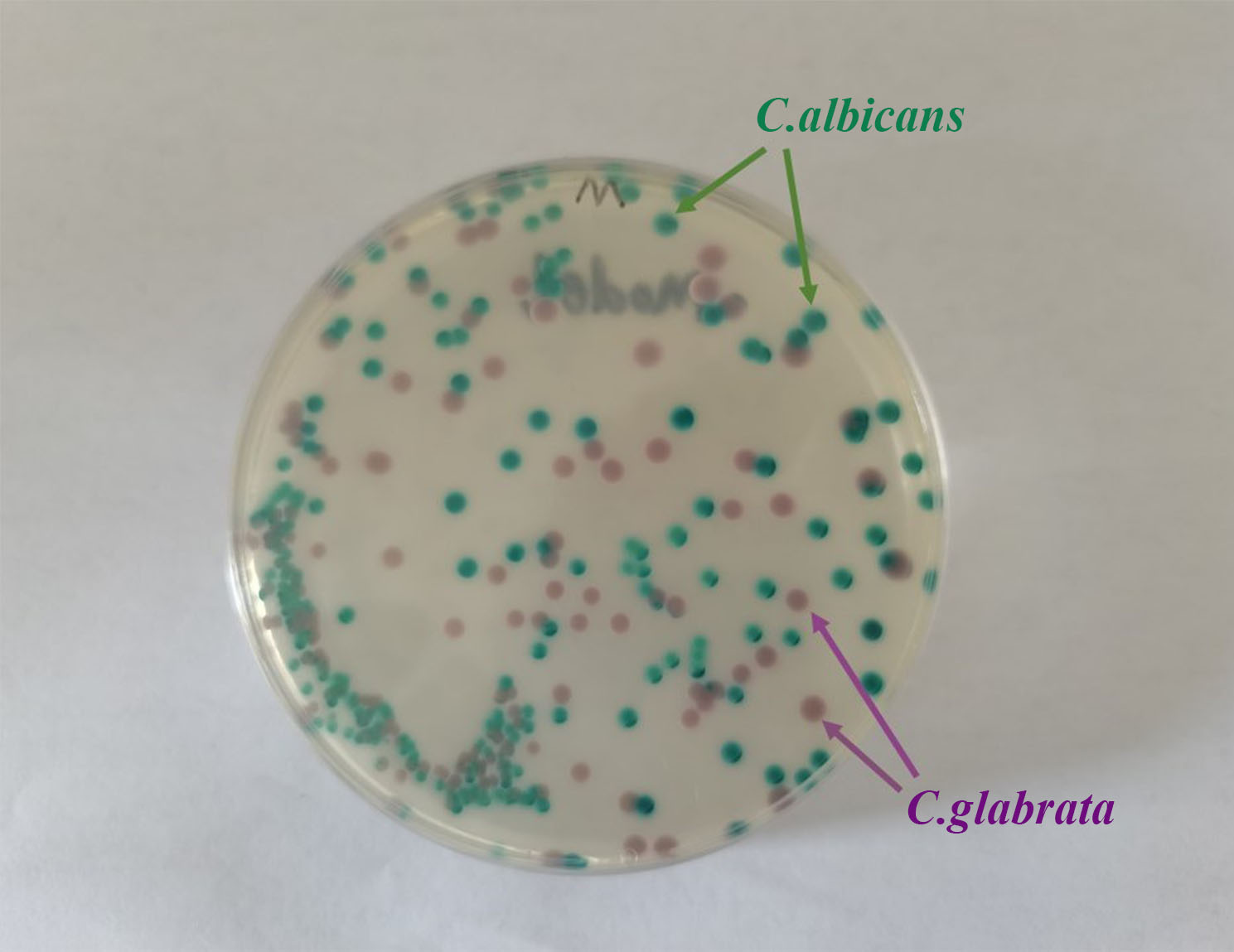


**Figure S5.** Fungal growth of tongue tissue homogenates on a Chromagar plate. Green: *C.albicans*. Lavender: *C.glabrata*.


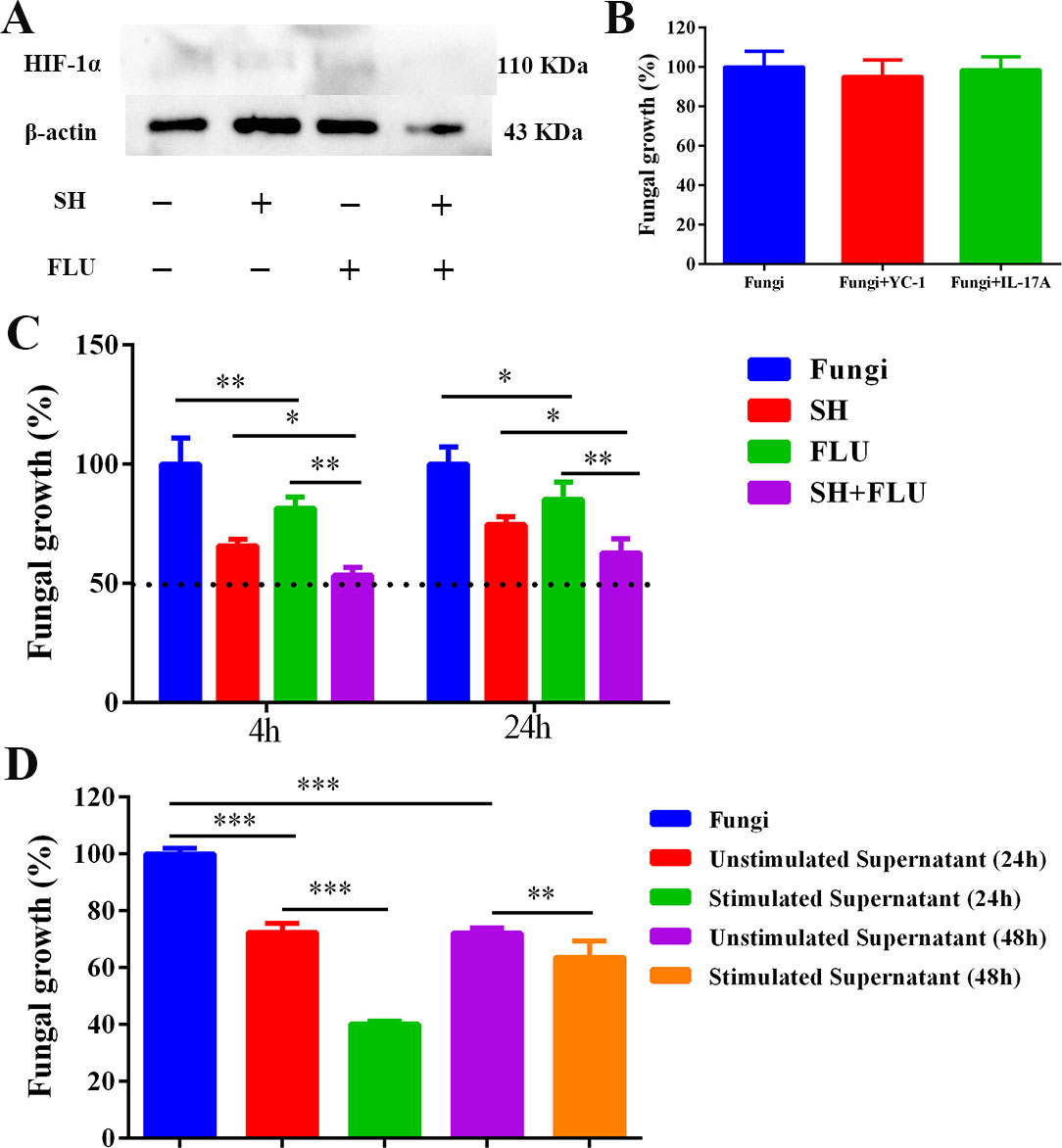


**Figure S6. A.** Representative western blot bands show that the drugs have no impacts on the expression of HIF-1α protein at 16 μg/mL SH and/or 8 μg/mL FLU in EC109. **B.** YC-1 (20 μM) and IL-17A (50 ng/mL) have no effects on the dual fungal growth in the presence of EC109 for incubation of 4 h. **C.** Mono- and dual- uses of 16 μg/mL SH and 8 μg/mL FLU inhibit the dual fungal growth after 4 and 24 h of incubations. **D.** Stimulated and unstimulated supernatants of EC109 by the mixed fungal cultures have diverse influence on the mixed fungal growth by cell counting in the presence of 16μg/mL SH and 8μg/mL FLU. EC109 cells are cultivated for 24 and 48 h, and then stimulated or unstimulated by the fungi for 4 h prior to harvesting supernatant. The fungal cells are resuspended in the harvested supernatant for another treatment of 4 h with the indicated concentration of SH and FLU before plate counting. * p<0.05, ** p<0.01, *** p<0.001. Fungi: *C. albicans* SC5314 and *C. glabrata* ATCC15126.
